# Age-dependent vestibular cingulate–cerebral network underlying gravitational perception: a cross-sectional multimodal study

**DOI:** 10.1186/s40708-022-00176-2

**Published:** 2022-12-21

**Authors:** Tritan J. Plute, Dennis D. Spencer, Rafeed Alkawadri

**Affiliations:** 1 School of Medicine, Department of Neurology, University of Pittsburgh, 3471 Fifth Avenue, LKB 8Th Floor, Suite 815.05, Pittsburgh, PA 15213 USA; 2 Department of Neurosurgery, Yale School of Medicine, New Haven, 06520-8062 USA; 3 Department of Neurology, Yale School of Medicine, New Haven, 06520-8018 USA

**Keywords:** Gravitational perception, Granger connectivity, Cingulate gyrus, Motor control, Intracranial EEG, Sensory integration, Aeronautics

## Abstract

**Background and objectives:**

The cingulate gyrus (CG) is a frequently studied yet not wholly understood area of the human cerebrum. Previous studies have implicated CG in different adaptive cognitive–emotional functions and fascinating or debilitating symptoms. We describe an unusual loss of gravity perception/floating sensation in consecutive persons with drug-resistant epilepsy undergoing electrical cortical stimulation (ECS), network analysis, and network robustness mapping.

**Methods:**

Using Intracranial–EEG, Granger causality analysis, cortico-cortical evoked potentials, and fMRI, we explicate the functional networks arising from this phenomenon's anterior, middle, and posterior cingulate cortex.

**Results:**

Fifty-four icEEG cases from 2013 to 2019 were screened. In 40.7% of cases, CG was sampled and in 22.2% the sampling was bilateral. ECS mapping was carried out in 18.5% of the entire cohort and 45.4% of the cingulate sampled cases. Five of the ten CG cases experienced symptoms during stimulation. A total of 1942 electrodes were implanted with a median number of 182 electrode contacts per patient (range: 106–274). The electrode contacts sampled all major cortex regions. Sixty-three contacts were within CG. Of those, 26 were electrically stimulated; 53.8% of the stimulated contacts produced positive responses, whereas 46.2% produced no observable responses. Our study reports a unique perceptive phenomenon of a subjective sense of weightlessness/floating sensation triggered by anterior and posterior CG stimulation, in 30% of cases and 21.42% of electrode stimulation sites. Notable findings include functional connections between the insula, the posterior and anterior cingulate cortex, and networks between the middle cingulate and the frontal and temporal lobes and the cerebellum. We also postulate a vestibular–cerebral–cingulate network responsible for the perception of gravity while suggesting that cingulate functional connectivity follows a long-term developmental trajectory as indicated by a robust, positive correlation with age and the extent of Granger connectivity (*r* = 0.82, *p* = 0.0035).

**Discussion:**

We propose, in conjunction with ECS techniques, that a better understanding of the underlying gravity perception networks can lead to promising neuromodulatory clinical applications.

**Classification of evidence:**

This study provides Class II evidence for CG's involvement in the higher order processing of gravity perception and related actions.

**Supplementary Information:**

The online version contains supplementary material available at 10.1186/s40708-022-00176-2.

## Introduction

The Cingulate Gyrus (CG) is a bilateral structure often divided into four main regions, including multiple subdivisions, each with complex and variable functions. Because of its varied connections, the cingulate is associated with different sensory, motor, and affective functions [1, 2]. It integrates memory and cognitive inputs to influence goal-directed behavior. The complexity and overlap of cingulate subregions lead to multiple epileptic auras, such as fear, psychosis, complex motor symptoms, and sensory distortions, making exact epileptic zone localization difficult [3–5]. Indeed, because of the relative rarity of cingulate epilepsy, posterior cingulate cortex (PCC) epileptic episodes are sometimes confused with seizures originating within the temporal lobe [3]. Besides, implanting the intracranial EEG (icEEG) electrodes needed for sampling does not come without risk [6]. Both the location and symptoms of cingulate epilepsy make conventional EEG localization suboptimal for adequate seizure localization. Thus, alternative methods must be used. Intracranial EEG (icEEG) or Electrocorticography (ECoG) enables a high-resolution mapping in both spatial and temporal dimensions; the technique reduces artifacts and explains precise connections.

A complex, multimodal set of integrative processes would occur within CG to contribute to learning and behavior. In this study, we report on a novel, stereotyped sensation of weightlessness/floating by direct CG 50-Hz electrical cortical stimulation (ECS) mapping in a consecutive series undergoing icEEG evaluations for the localization of epileptic focus. We also studied functional relationships with other brain regions using fMRI, icEEG, and cortico-cortical evoked potentials (CCEP) and discussed the findings contribution to the knowledge of CG.

A comprehensive understanding of cingulate functional connectivity will aid clinicians in better recognizing the symptoms of epilepsy. Research may uncover promising areas of neuromodulation for treating complex chronic disorders affecting balance perception. Today, gravity is no longer only explained by Newtonian mechanics. Its alternations are experienced in real-world space exploration and identifying networks that influence its human perception and modulation may relate to the practice of aeronautics.

## Materials and methods

### Patients and EEG data acquisition

We screened consecutive cases with refractory epilepsy undergoing icEEG monitoring at Yale Comprehensive Epilepsy Center between 2013 and 2019 for localization of refractory seizures in which CG sampling was indicated. The primary outcome of the study is defined as the clinical response observed and brain regions functionally connected to the areas of interest. We excluded cases, where CG’s mapping overlapped with the seizure onset zone.

### Implantation of electrodes and EEG data acquisition

Multiple intracranial EEG electrodes, including platinum disk subdural grids 4 mm in diameter, multiple strips, and depth electrodes 2.3 mm (AdTech Medical, Racine, WI, USA), were placed according to a multi-disciplinary surgical conference discussion. icEEG was recorded on a 256–512 channel video-icEEG long-term monitoring device (Natus Medical Incorporated, CA). EEG recordings were referenced to an electrode implanted in the diploic space using an active ground reference setup. The EEG electrode sampling frequency was 1024 Hz or higher. Data were collected with a hardware high-pass filter of 0.1 Hz. The passive network analyses were performed in 15–60 min of artifact-free, relaxed awake records.

### Contact coregistration using preoperative MRI

We used pre- and post-implantation magnetic resonance imaging (MRI) data and post-implantation computerized tomography to coregister each electrode contact. Contact sites were assigned a pre-implantation location on a three-dimensional triangular mesh model of the patient’s cortex via tools available through BioImage Suite software (v3.0; New Haven, CT, USA). All contacts were assigned to both lobes (frontal, parietal, temporal, occipital, or insular) and sublobes using anatomical landmarks.

### Cingulate anatomy and subdivisions

The technique followed major known divisions inspired by current knowledge of histopathological distinctions [7]. The technique followed major known divisions. We approximated the radiologic sectors of the cingulate divisions in the coronal plane passing through the anterior/posterior commissure VCA and VCP lines [8], understanding that the histopathological delineations are less well-defined with a variable overlap in the Anterior Cingulate Cortex (ACC) around the VCA line. VCP delineates the division between the PCC and the rest of the CG. Whereas the VCA dissects the Middle Cingulate Cortex (MCC) into two regions. See Additional file 1: Fig. S1.

### Functional mapping by high-frequency stimulation

Electrical stimulation mapping was completed extra-operatively alongside continuous ECoG recordings that routinely occurred in the last 1–2 days before surgical electrode removal. A Nicolet Cortical Stimulator (Natus Medical Incorporated, CA, USA) was used for current-controlled stimulation. 1–5 s 50-Hz trains of bipolar, biphasic, and rectangular waveforms of 0.3-ms pulses were delivered to patients for stimulation mapping. Patients were monitored during a stepwise increase in current by 0.5 to 1 mA and asked to report aberrant sensations or seizure auras. Two sham simulations were used to confirm positive subjective reports, and the current increase ceased if seizures or after-discharges occurred, or 12 mA amperage was reached. Furthermore, the examiners classified responses according to a 4-point classification for the sensory-motor responses [8]. For others, we used an established semiology-based classification [9].

1. Type of response: i. Motor: a. Tonic b. clonic c. automotor d. complex motor, or e. hypermotor. ii. Sensory: a. Tingling b. numbness c. temperature d. complex. iii. Visual: simple, complex iv. Auditory: simple, complex v. Psychic: class, simple, modifiers.
2. Part of the body involved a. hand, b. arm, c. axial, d. face, e. tongue, f. leg.
3. Side(s) involved a. Ipsilateral b. contralateral to the site of ECS c. axial/central or d. bilateral.
4. ECS parameters correlated with the elicitation of the symptom.

### Cortex mesh generation

**Fig. 1:**
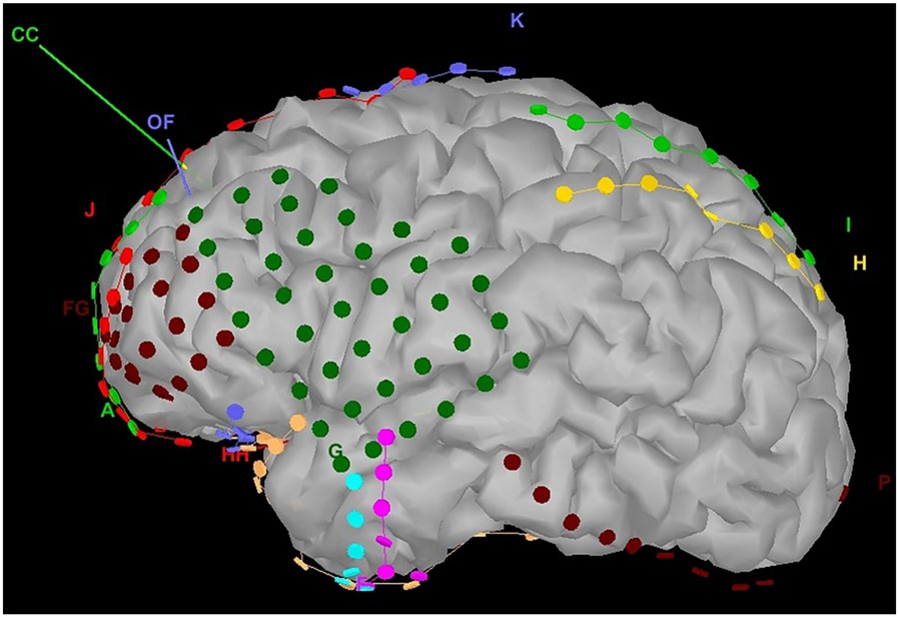
Reconstruction of electrodes on a mesh of a preimplantation 3-D reconstruction of the patient's own MRI (case 7)

Preoperative MRI scans were imported to the Brainsuite or BrainVisa program to generate a unique three-dimensional cortical mesh. Files were then exported into Brainstorm3 (https://neuroimage.usc.edu/brainstorm) to perform coregistration with electrodes and to generate subsequent cortical activation maps. Following individual reconstructions, cortices were downsampled to 10,000 vertices per case to simplify computation and ensure proper coregistration of electrode contacts and EEG data fidelity (Fig. 1). We made the electrodes and cortex reconstructions available for review in Additional file 2: Fig. S2.

### Multimodal network analyses are

#### Rationale

While the EEG has excellent temporal resolutions, relying on intracranial EEGs may result in so-called cortical myopia, since subcortical and white matter potentials are rarely recorded or analyzed. fMRI and, while limited in temporal resolutions, provide excellent insight into subcortical structures. As the floating sensation cannot be perceived naturally at the bedside—we relied on the analysis of seed-based functional connectivity. The cortical evoked potentials represent multisynaptic local field connectivity uniquely beyond anatomical (bundles) or functional (co-activation) measures. Some view it as a more accurate representation of common-sense connectivity or effective connectivity, but it requires current injection. Hence, we believe our multimodal mapping approach provides together an incomparable spatial and temporal view of networks underlying observed phenomena.

Other methodological details are available in Additional file 3.

### Statistical analysis

We carried out statistical assessments of correlations using post hoc Pearson correlation analysis. We employed Benjamini–Hochberg procedure to correct for multiple comparisons. The pre-test *p* value was 0.01. The extent of Granger connectivity values was compared to other clinical variables (IQ, age at onset, age at presentation, and duration of epilepsy). The functional relationship were explored both per absolute connectivity values and regional/subregional relationships. We used maximum connectivity values and number of activated pixels as a surrogate indicator of the strengths and regions of activation.

### Data availability

We can make these data available upon reasonable inquiry.

### Standard protocol approvals, registrations, and patient consents

The study received approval by an ethical standards committee at Yale University protocol # 1408014451. This study does not include live vertebrates or higher invertebrates. The IRB determined that participant consent was not required in this retrospective data analysis.

## Results

### Demographics

Fifty-four icEEG cases from 2013 to 2019 were screened from a single institution, with 40.7% of cases sampled from the cingulate gyrus and 22.2% using bilateral sampling. 18.5% of the entire cohort and 45.4% of the cingulate cases underwent high-frequency electrical stimulation. Five of the ten CG cases experienced symptoms during stimulation.

Consult Table 1 for a summary of the demographics. Five cases were female. The median age at the onset of epilepsy was 16 years (range: 5–47). A total of 1942 electrodes were implanted with a median number of 182 electrode contacts per patient (range: 106–274).

**Table 1:** Demographic information for subjects included in the study

Case	Gender	Age at onset and duration (years)	Typical Seizure	Frequency per month	MRI	Language fMRI	ictal EEG onset and patterns
*1*	F	32 (9)	Somatosensory Aura > Autonomic	10 +	Left frontal T2 increased signal	Left	Temporal
*2*	F	5 (15)	Somatosensory aura > Bilateral Tonic Leg > dialeptic > Left arm/leg clonic	45	Left posterior parietal cortical dysplasia and thickening	Left	Parietal
*3*	F	5 (9)	Neck extension, bilateral arm extension, seconds to a minute, clusters in early morning	600	Stable diffuse enlargement of the lateral and third ventricles with periventricular gliotic change	Not lateralized	N/A
*4*	F	26 (18)	Psychic Aura > bilateral leg/arm hypermotor > dialeptic (staring to left) > left arm clonic jerk > left arm tonic extension	1─2	Postoperative changes consistent with anterior cingulectomy	Left	Frontal
*5*	M	22 (1)	Abdominal aura	Sporadic	Unremarkable	N/A	Temporal
*6*	F	12 (7)	Pyschic aura (Panic Attack) > Autonomic	9	Unremarkable	Left	Temporal (anterior, middle)
*7*	M	12 (25)	Bilateral arm and leg tonic with extension > generalized tonic–clonic	5	Left hippocampal malrotation	Left	Frontal with rapid bi-synchrony within 1.5 s
*8*	M	20 (5)	Aura of tingling sensation that starts in the bilateral hands and marches upward toward the arm. The sensation of déjà vu	5	Normal	Right	Temporal
*9*	M	47 (9)	Sensation of left lower face twitching and lightheadedness + / − (Rare) progression to generalized tonic–clonic	10 +	Left frontal subcortical microhemorrhage Right superior temporal Thinning of the right frontal face region	Left	Centro-temporal
*10*	M	14 (4)	Left head and eye versive > generalized tonic clonic	90 +	Right frontal focal cortical dysplasia	Right	Fronto-Temporal

Males are noted as M and females as F

The electrode contacts sampled all major cortex regions, including depth electrodes within the cingulate, hippocampus, insula, and amygdala. Cortical sampling represented a percentage of total contacts: 53.7% frontal, 25.6% temporal, 10.5% parietal, 6.2% occipital, and 4% insula. Sixty-three contacts were within the cingulate cortex. Of those, 26 were chosen for stimulation; 53.8% of the stimulated cingulate contacts produced positive responses, whereas 46.2% produced no observable responses. Only 28.6% of ACC cases exhibited symptoms, whereas 83.3% of MCC cases did, and the singular PCC case responded to stimulation. One pair was excluded, since only one contact fell onto the cingulate sulcus outside CG proper, resulting in an out-of-body experience (case 4).

### Functional findings

Of the stimulated pairs, 21.4% elicited vestibular responses, while 28.6% generated motor symptoms, 14.3% sensory, and 42.8% had no perceived responses. One site had mixed motor/sensory symptoms. The median current intensity for positive findings was 5 mA, interquartile range (4–6) mA. Vestibular symptoms manifested as a perceived loss of gravitational sense resulting from stimulation of ACC and PCC. Motor auras manifested in various ways, but a grouping of MCC provoked motor responses associated with tongue movement and swallowing or contralateral arm posturing and negative motor responses. The MCC stimulation elicited prickly sensation in the bilateral knees in one case and visceral epigastric sensations in another. All other stimulation sites produced no perceived symptoms.

### Intracranial EEG-based cortical connectivity findings

**Fig. 2:**
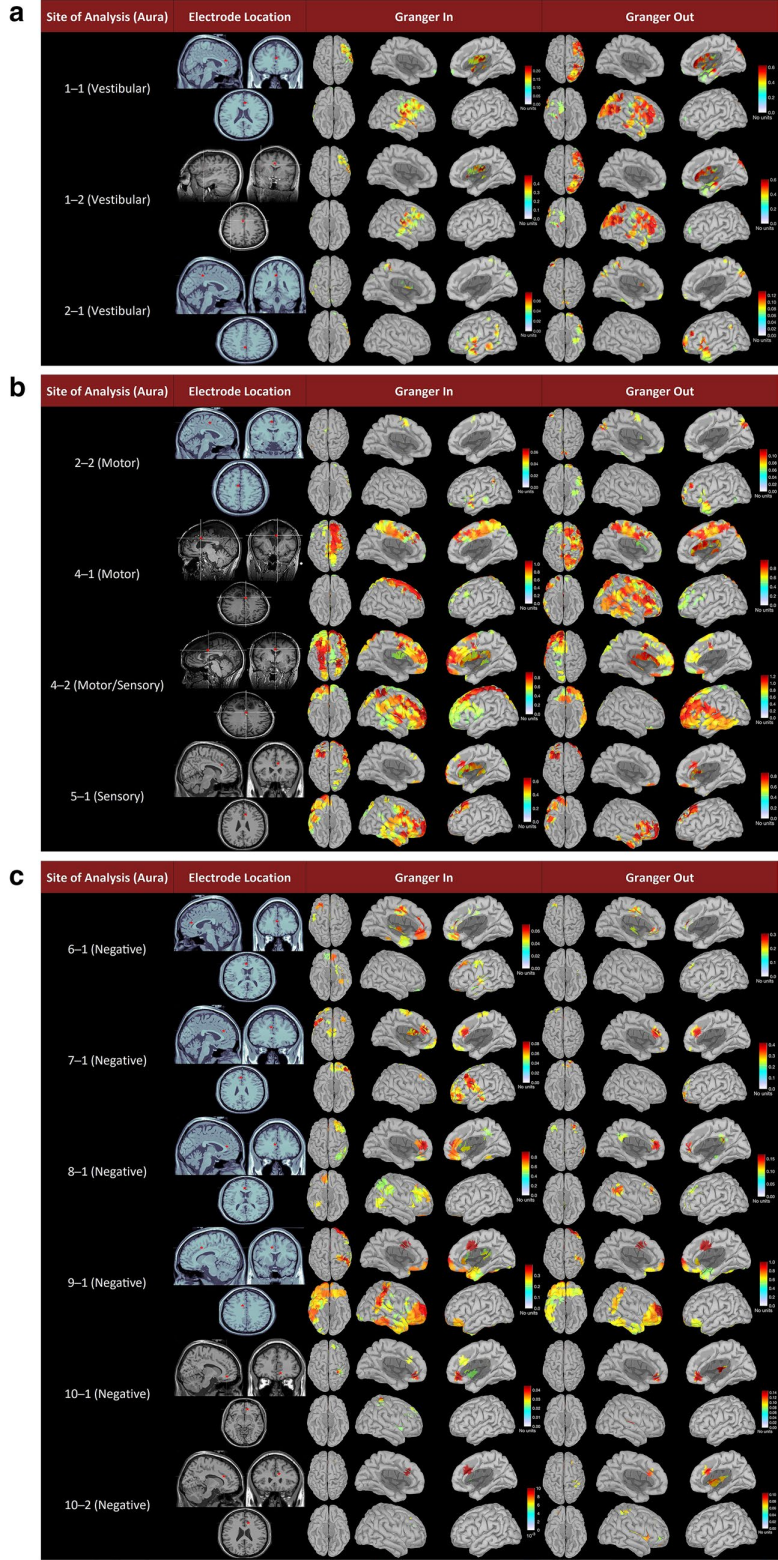
Recorded functions, stimulation sites, and Granger connectivity findings in different plains and accompanying unitless scales

Seven of the 13 Granger analyzed sites localized to the right cingulate gyrus; however, Granger causality showed consistent bilateral connectivity in all but one subject, displaying an ipsilateral response (Fig. 2, Table 2).

**Table 2:** icEEG Granger analysis results for each stimulated contact

Stimulated contact	Baseline IQ	Function (Y/N)	Function category	Site of stimulation (R/L)	Site of Stimulation	Sampling	Granger-In	Granger-Out	Max Granger-in	Max Granger-Out
*1─1*	84	Y	Vestibular	L	ACC	Bi/R +	Bi pref R	Bi pref R	0.2	0.6
*1─2*	=	Y	Vestibular	R	ACC	Bi/R +	Bi pref R	Bi pref R	0.4	0.6
*2─1*	105	Y	Vestibular	L	PCC	L	Bi pref L	Bi pref L	0.06	0.12
*2─2*	=	Y	Motor	L	MCC	L	Bi pref L	Bi pref L	0.06	0.12
*3─1*	61	Y	Motor	L	MCC	L	N/A	N/A	N/A	N/A
*4─1*	123	Y	Motor	L	MCC	Bi	Bi pref R	Bi pref R	1	0.8
*4─2*	=	Y	Motor/Sensory	R	MCC	Bi	Bi pref R	Bi pref L	0.8	1.2
*5─1*	109	Y	Sensory	R	MCC	Bi/R +	Bi pref R	Bi pref R	0.5	0.8
*6─1*	67	N	Negative	R	ACC	Bi	Bi pref L	Bi pref L	0.06	0.3
*7─1*	106	N	Negative	L	ACC	Bi/L +	Bi pref L	Bi pref L	0.08	0.4
*8─1*	94	N	Negative	L	ACC	Bi	Bi pref R	Bi pref R	0.8	0.15
*9─1*	120	N	Negative	R	MCC	Bi/R +	Bi	Bi pref L	0.3	1
*10─1*	92	N	Negative	R	ACC	Bi/R +	R	R	0.02	0.014
*10─2*	=	N	Negative	R	ACC	Bi/R +	R	R	0.0015	0.002

IQ = Intelligence Quotient. Sampling is indicated as either bilateral (bi), left (L), right (R), bilateral with right dominance (bi/r +), or bilateral with left dominance (bi/l +). Stimulation sites include the anterior cingulate cortex (ACC), the middle cingulate cortex (MCC), and the posterior cingulate cortex (PCC). Granger-in and -out values are expressed as unitless correlation coefficients and are assigned signal dominance of bilateral (bi), left (L), right (R), bilateral with left preference (bi pref L), or bilateral with right preference (bi pref R)

Irrespective of the elicited symptoms, there were consistent ACC links to the superior frontal, middle frontal, middle temporal, inferior temporal, precentral, and postcentral gyri, and occasional connections from the ACC, PCC, orbitofrontal insular cortices, and the superior occipital gyrus. ACC stimulated sites' output localized to the superior and middle frontal and temporal regions, inferior temporal, and parietal gyri. Less frequent connections to the occipital lobe, the insular cortex, ACC, PCC, and the orbital frontal cortices were observed. Furthermore, MCC exhibited a robust connectivity profile including strong output connections in several cases, and inputs from most of the frontal and temporal cortex with significant connections to the inferior frontal, peri-central regions, ACC and PCC. The superior occipital, lateral occipital, superior parietal, and inferior parietal gyri also made functional connections with the MCC. Notable regions of MCC’s output included the frontal and temporal lobes, superior occipital gyrus, inferior frontal region, and superior and inferior parietal gyri.

The PCC exhibited weaker connection values than ACC; however, functional connections arose from the superior and middle temporal gyri, and pre- and post-central gyri were observed. Likewise, outputs from PCC to precentral, lateral occipital, superior temporal, and middle temporal gyri were notable.

### CCEP findings

Cortico-cortical evoked potential (CCEP) signals overlapped with the functional connections corroborated by Granger causality at the five sites when performed: four in ACC and 1 in PCC. All five sites exhibited bilateral connectivity; however, only one showed strong bilateral activation. The other four displayed a strong predilection for one hemisphere—typically the ipsilateral hemisphere—although one case exhibited contralateral connectivity.

**Fig. 3:**
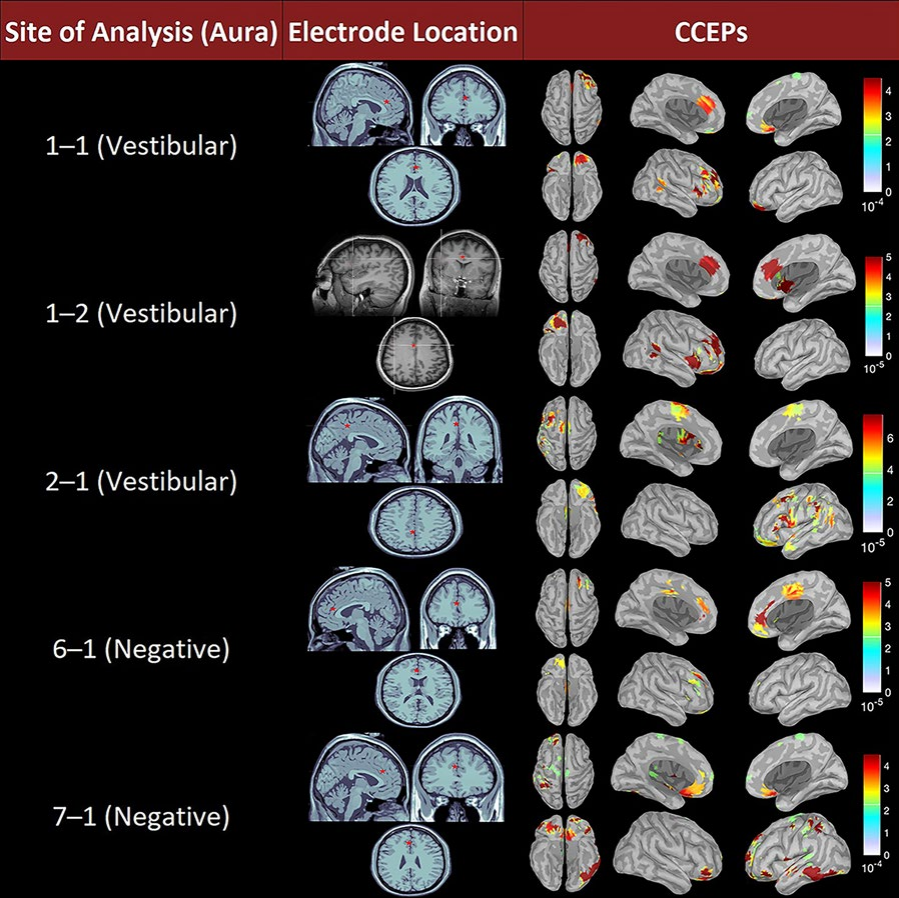
CCEP stimulation sites and average record evoked responses as root mean square as μV^2^/s

There were notable functional ACC connections across most frontal and temporal lobes besides the parietal lobes. The insular cortex, ACC, and MCC displayed the most robust connections. The superior, middle and inferior frontal gyri; superior and middle temporal gyri; and inferior parietal gyrus were associated with CCEP data and can be visualized in Fig. 3. Like Granger connectivity’s findings, the connectivity profile did not appear to be function specific.

### fMRI findings

Functional MRI connectivity maps were generated for nine stimulation sites: four from both ACC and MCC and one from PCC. Granger data coincided with fMRI networks. fMRI data from contact seeds were bilateral, with every site exhibiting a subcortical activation. Increased brainstem co-activation was present but was weaker with the MCC, presenting with robust cerebellar activation instead.

**Fig. 4:**
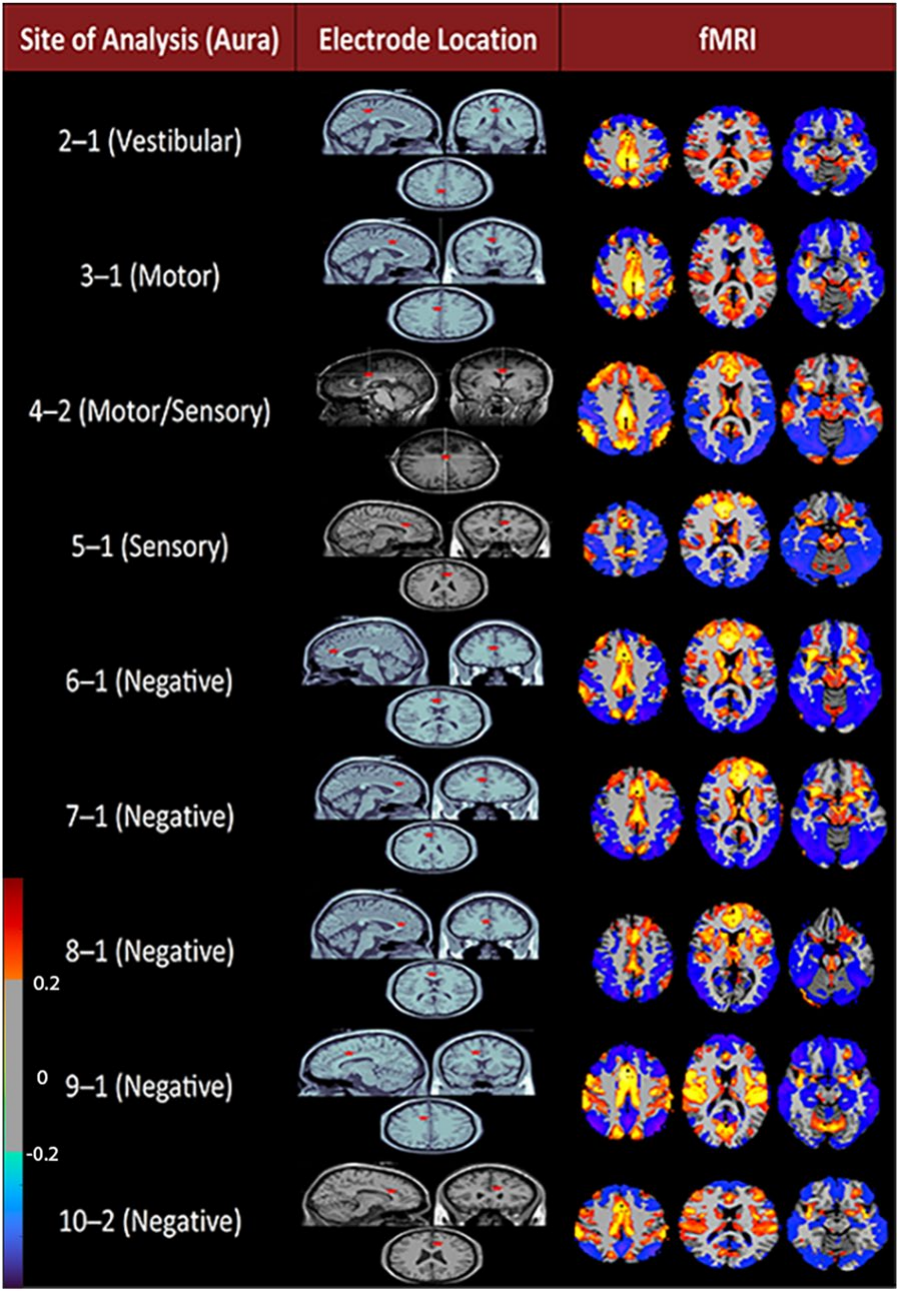
fMRI correlation maps

Functional-MRI analysis of ACC sites displayed increased frontal and parietal lobe connections and increases in the insular cortex and brainstem co-activity. Respectively, ACC sites consistently exhibited increased frontal lobe co-activity across cingulate and orbitofrontal cortex regions, whereas other areas of activation demonstrated some variation between sites. MCC sites also generated co-activation in the frontal, insular and parietal lobes with general cingulate activation; however, other areas of activation include the cerebellum and occipital lobe. The brainstem was activated in MCC sites, albeit lesser than in ACC. Increased frontal and parietal lobe signaling are present in the PCC site, and cingulate activation is strong; however, brainstem activity is localized to the superior colliculus only; insular activity is less pronounced, whereas the orbitofrontal cortex showed a decrease in activity. Of note, brainstem activity was not increased at this site. fMRI data can be viewed in Fig. 4.

### Cingulate connectivity with age

As the function subtype was not the main foundation for the connectivity models. We then investigated the relationships between connectivity and different clinical features. The multiple variables tested included IQ, duration of epilepsy, age at presentation and onset of disease, and seizure frequency. Only age at presentation correlated with the degree and extent of site connectivity to other brain regions, *r* = 0.82, p 0.0035, corrected for multiple comparisons. Meaningful comparisons showed an expected correlation between the ages of onset and presentation, *r* = 0.865, p 0.0003. A non-significant trend has been observed between Granger maximum and the age of onset. The relevant data are presented in Table 2 and Fig. 2.

## Discussion

### Highlights

- Our study reports a unique perceptive phenomenon of a consistent subjective sense of weightlessness/floating sensation triggered by anterior and posterior CG stimulation previously underreported in the capacity.
- Our findings highlight an extensive hieratical cortical–subcortical network, and in particular premotor insular and cerebellar involvement in gravity perception.
- An interesting result is a correlation between age at presentation, the extent of Granger network activity and maximum values, and no other clinical variables. This may indicate that at least certain CG subregions maturate as humans age. Future prospective controlled studies may elucidate different subregions' contributions to this phenomenon.

### Vestibular cingulate network

Our study reports a unique perceptive phenomenon of a consistent subjective sense of weightlessness/floating sensation triggered by anterior and posterior CG stimulation, with 30% of cases and 21.42% of electrode stimulation sites evoking this perception. This finding is underrepresented in literature [10] and has not been reported in earlier comprehensive efforts to map the vestibular cortex in humans. [11], Among others, highlighting perhaps the importance of enhancing testing paradigms of functional mapping that are biased to detect sensorimotor and linguistic functions by design. Taken together, reasonable to assume that both anterior and posterior cingulate divisions play a role in the tertiary processing of vestibular processing and the perception or change of weightlessness along the primary and secondary vestibular cortices in the insula and temporoparietal cortices, but at higher order levels, such as adaptation, learning, integration, and reaction. The vestibular system is an ancient evolutionary sensory modality [12] that begins in the inner ear and travels to several nuclei within the brainstem. [11] Because of their role in maintaining balance and encoding rotational perception, the vestibular nuclei have many complex and far-reaching connections.

Our connectivity data suggest vestibular inputs reach the cingulate via the insular cortex. Moreover, a recent study using functional connectivity in epilepsy patients has shown that an area of the CG involved in the awareness of self-motion has established networks to the insular cortex—an area thought to act as the primary vestibular sensory cortex [13]. Likewise, other functional connectivity studies have provided convincing evidence for vestibular–cingulate interaction via vestibular dysfunction studies. Indeed, reduced connectivity between the anterior cingulate cortex and the posterior insula has been associated with vertigo, suggesting (mis)-communication between the areas [14].

The interplay between the two cingulate ACC and PCC regions is complex, given the similar connectivity profiles of the three sites. We hypothesize that the role of CG in this setting is hierarchical (ACC) and integrative (ACC, PCC), akin to its well-established role in behavioral modification and learning. Similar to that observed around the multilayer processing pathway from S2 to the posterior insula in hierarchical sensory processing. [15] Interestingly, PCC’s fMRI connectivity showed specific activation of the superior colliculus—implicating the cingulate in behavioral motor control of ocular movement. Indeed, evidence exists suggesting that the cingulate integrates cognitive, motor, vestibular and other sensory information to influence eye movement in lower mammals. [16]

### Age-related Granger correlation

An interesting result is the correlation between age at presentation, the extent of Granger network activity and maximum values, and no other clinical variables. Former studies cite that ACC gray matter volume is reduced in children with disruptive behavior disorders than in healthy controls [17]. Healthy older adults showed decreased anisotropy in specific cingulate networks compared to younger comparisons [18]. Our findings are compatible with the known CG’s role in learning and behavior [2]. While evidence suggests that cingulate functional connectivity matures over many years, further research and larger sample size to appraise many covariates are needed to decide whether our reported correlation can be attributed to developmental causes. This finding could not only redefine the traditional plasticity paradigm within the CG; it may likewise apply to other higher cognitive centers. Our findings defy the old tradition of an early plateau in brain development and are consistent with recent histopathological findings of studying Von Economo neurons in humans, and the concept of ongoing plasticity at the synapse level. These cells are believed to have the unique role of associating emotions and actions, just like manual dexterity depends on close connections between contiguous somatosensory and motor areas. Interestingly, they are many more in adult humans than in infants, suggesting that their proliferation parallels the accumulation of affect-encoded knowledge across the lifespan.

### Functional symptom interpretation and network analysis

The perceived symptoms can be grouped into vestibular responses following ACC and PCC stimulation and sensorimotor responses following MCC stimulation. The significant number of negative responses is not surprising, as it has been a consistent finding by others too [19]. For example, in an earlier study, stimulating the posterior cingulate cortex did not produce any observable responses, even though it is one of the most active parts of the brain in the metabolic sense. This emphasizes the importance of developing new clinical mapping models to assess findings in addition to those presently linked only with significant deficits after surgery [20]. Our results are compatible with the known role of MCC in multimodal sensory integration and goal-directed, complex motor output [1] thus, the observation of complex motor (speech and swallowing) behavior and sensory phenomena following MCC stimulation is unsurprising. Together with the MCC, which generated complex behaviors in the face and upper extremities—an expected response from a higher motor center. In the past, there have been sentiments suggesting that the cingulate is an inactive region in the functional sense; however, our findings add to a growing literature that argues that the sampling and testing paradigms implemented in icEEG do not meet the complexity of CG’s functionality. The cingulate has been associated with motor deficits such as akinetic mutism [21] and many autonomic, affective and somatosensory deficits [19]. According to our connectivity data, bilateral CG functions in synergy with both hemispheres, to a variable extent, so profound dysfunction is only associated with bilateral injury. These data, along with the usually reported undersampling in icEEG ^33^, highlight the need for a more anatomy-functional-minded approach to sampling CG in the case of surgical epilepsy.

However, CG connectivity maps were primarily function-independent. This may represent a unique robust connectivity profile that parallels the hierarchical interagative processing that occurs within, or it is possible that certain specific connections are underestimated by the resolutions of icEEG though excellent and practical yet not as precise as single- and multi-unit recordings. That said, the sites exhibiting vestibular symptoms had a lesser degree of “within-CG” activation and is probably consistent with unique localized processing observed here that is different from the more common cingulate parts reported within the cingulate as hierarchal tertiary motor programs. ACC further displayed a robust brainstem activation consistent with its role in somatic and autonomic output [1]. PCC networks showed overlapping findings with ACC—with more predilection to temporal regions; thus, it is no surprise that PCC seizures masqueraded as temporal lobe epilepsies [3]. The MCC networks, as expected, exhibited main outputs to the motor and sensory regions and inputs from these same areas, as well as ACC and PCC. When joint with fMRI data showing widespread frontal cortex activation, these data align with the MCC’s role in adaptive motor behavior and cognitive appraisal [22].

### Clinical potential

Given the recent interest in neuromodulation, our findings apply to the clinic. Several neurological deficits have been linked to vestibular malfunctioning [14, 23–25]. Because the Cingulate Gyrus is involved in vestibular processing, this region provides an inviting potential target for treating several disorders. Clinicians may soon control debilitating symptoms safely by explaining the connections underlying network-wide dysfunction. The study of noninvasive modulatory effects of transcranial stimulation could further extend to aerospace exploration, where there has been evidence of electrophysiological changes in unearthly gravitational environments ^35^. Likewise, future treatment could apply to other at-risk populations to prevent, rather than treat, issues arising from vestibular malfunction.

### Methodological limitations

The cross-sectional approach mitigated the typical retrospective design limitations. To decrease the error in coregistration estimated at 7 mm for the best-case scenario, we considered the case's anatomy one at a time [26]. We used sham stimulations at every positive response site to ensure positive feedback was not coincidental, since electrical stimulation has known imperfections with current shunting. However, it remains the leading method of mapping human brain functions [27]. It is not easy to decide the extent of functional contributions and correlation of a region based on noninvasive methods. We are, however, confident in a multimodal approach revealing excellent collective resolutions ^36^. Even though the areas included did not fall within the seizure onset zone, we realize the imperfections of grading a contact location on the wide range between normal and abnormal in a cohort of epilepsy patients. That said, it is considered acceptable to use the seizure onset, and highly irritative zone to determine abnormality in otherwise normal physiological brain regions [28]. Cingulate maturation is not synonymous with increased connectivity, however, as healthy older adults have been reported to show decreased functional anisotropy in specific cingulate networks when compared to younger comparators [29]. Electrical noise of 60–120 Hz cycles was bypassed using a bandpass of 70–115 Hz in a high-gamma Granger Causality analysis. Due to its design, Granger Causality avoids issues with volume conduction and correlation/coherence inflation.

## Conclusion

We showed a high incidence of vestibular symptomatology and novel findings of weightlessness, and lack of a sense of gravitational perception following stimulation of the cingulate gyrus, in particular, the ACC and PCC. These locations show strong connections with the cortical and subcortical (pre) motor systems. The significant correlation between the extent of connectivity with other brain regions and age highlights the possibility that cingulate circuits maturate as human learning and development mature. Finally, these mapped networks may prove salient in the modulation and prevention of many vestibular disorders, making this work an essential preclinical step toward new treatment paradigms.

## Supplementary Information

**Additional file 1: Figure S1.** Map of major divisions of the cingulate gyrus and relation to VCA and VCP lines. PC = Posterior commissure, AC = Anterior commissure.

**Additional file 2: Figure S2.** Reconstruction and implantationof electrode contacts on 3D mesh reconstruction of preoperative MRIs. To visualize different electrode arrays implanted, color coding is arbitrary.

**Additional file 3.** ECoG processing and network analysis.

## Abbreviations

The ACC: Anterior cingulate cortex
CCEP: Cortico-cortical Evoked Potentials
CG: Cingulate gyrus
ECoG: Electrocorticography
ECS: Electrical cortical stimulation
icEEG: Intracranial EEG
MCC: The middle cingulate cortex
PCC: The posterior cingulate cortex
