# Supplementary figures and images for: Age-dependent vestibular cingulate–cerebral network underlying gravitational perception: a cross-sectional multimodal study
Source: Brain Inform. 2022 Dec 21;9(1):30. doi: 10.1186/s40708-022-00176-2 (PMC9772366; doi:10.1186/s40708-022-00176-2)

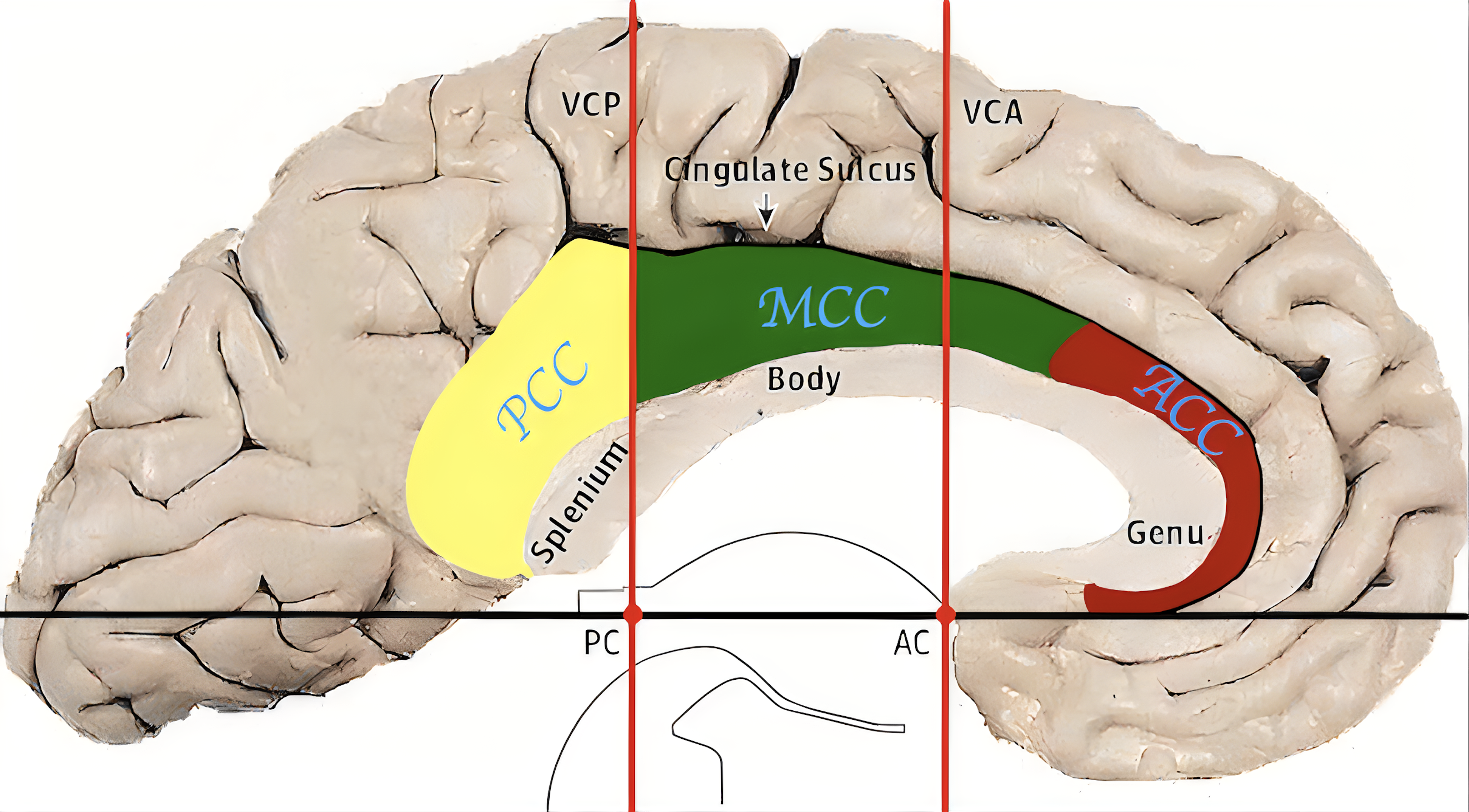

Supplement: Supplementary file 1 — Additional file 1: Figure S1. Map of major divisions of the cingulate gyrus and relation to VCA and VCP lines. PC = Posterior commissure, AC = Anterior commissure. [file 40708_2022_176_MOESM1_ESM.png]

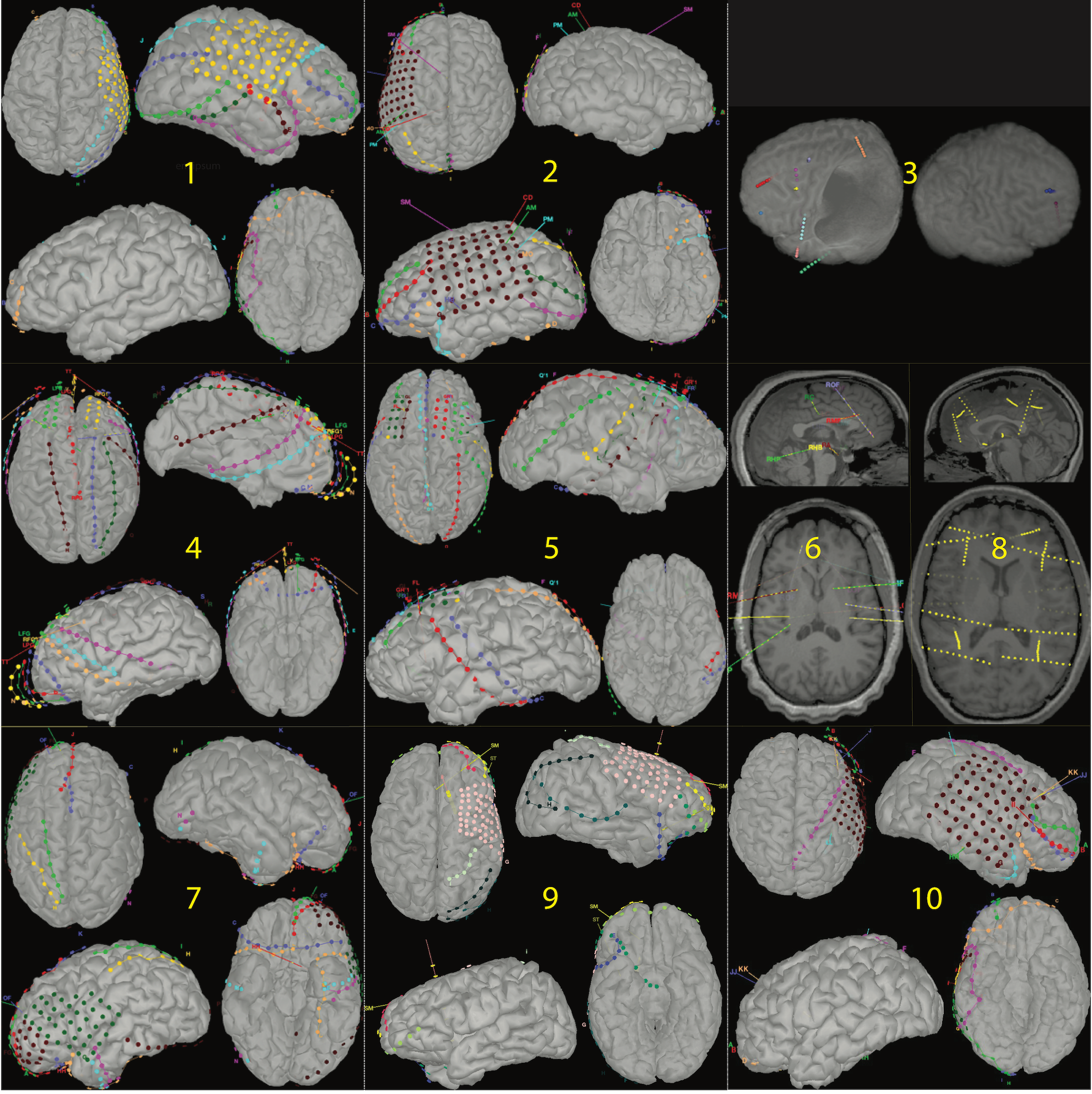

Supplement: Supplementary file 2 — Additional file 2: Figure S2. Reconstruction and implantationof electrode contacts on 3D mesh reconstruction of preoperative MRIs. To visualize different electrode arrays implanted, color coding is arbitrary. [file 40708_2022_176_MOESM2_ESM.tiff]
