# Supplementary material for: Age-dependent vestibular cingulate–cerebral network underlying gravitational perception: a cross-sectional multimodal study
Source: Brain Inform. 2022 Dec 21;9(1):30. doi: 10.1186/s40708-022-00176-2 (PMC9772366; doi:10.1186/s40708-022-00176-2)
Supplement: Supplementary file 3 — Additional file 3. ECoG processing and network analysis. [file 40708_2022_176_MOESM3_ESM.docx]

ECoG Processing and Network Analysis

Intracranial EEG data were scanned for artifacts that may obscure statistical analysis and processed before being imported into MATLAB. Within the program, the ECoG data was preprocessed.

The analysis was performed on two segments of resting awake with the patient resting comfortably in bed, mostly with eyes closed. The trials were then averaged after verifying reproducibility with correlation co-efficient between two trials >0.9. The analyses were performed on time-series of filtered EEG data ≥ 70-115 Hz using 4^th^ order Butterworth filter in agreement with previous methodologies to avoid 60-120 Hz electrical source cycle noise as well. ^1^ High-frequency oscillations have been exploited as reliable indicators of brain regions interfacing at the local field level. ^1 2^ The metric enables the estimation of apparent directionality and strength of a connection. It acts as a measure of linear dependence, which tests whether the variance of error for a linear autoregressive model estimation of a signal (X) can be reduced when adding a linear model estimation of a second signal (Y). If this is true, signal Y has a Granger Causal effect on the first signal X. It is important to note that though the directionality of Granger Causality is a step closer to measuring effective connectivity than symmetrical measures, it should still not be confused with “true causality”. Granger values are already normalized because Granger causality is a ratio of variances (so affine transformations of any signal have no effect on Granger causality). We applied Granger causality on the filtered signal in a linear bivariate and stationary model defined in both the time and spectral domain. If x represents a signal that can be modeled using a linear autoregressive model estimation in the following two ways:

$x= \sum_{k=1}^{p} [A_{k}x(t-k)]+e1$ (1) and $x = \sum_{k=1}^{p} \left[ A_{k} x\left( t-k \right)+B_{k}y\left( t-k \right) \right]+e_{2}$ (2)

Where *p* represents the amount of past information that will be included in the prediction of the future sample and is called the model order; in these two equations, the first models x using the past (and present) of only itself, whereas the second includes the past (and present) of a second signal y. Note that only past signals measures are taken into account ($\geq1).$ The model ignores simultaneous connectivity, making it less susceptible to volume conduction. ^3 4^

Phase locking is a fundamental concept in dynamical systems and in analyzing nonlinear, chaotic, and non-stationary systems. Since the brain is a nonlinear dynamical system, phase locking is appropriate for quantifying interaction. A more pragmatic argument for its use in studies of local field potentials.

CCEP methodology/single pulse stimulation

The study was conducted in a resting state shortly after patients resumed complete dose of their anti-seizure medicines at the end of the evaluation. Using postoperative MRI image reconstructions to confirm positioning, two adjacent bipolar contacts within the anterior cingulate gyrus delivered constant current square waves of 0.3 milliseconds to the region at a frequency of 1 Hz with alternating polarity. Starting at 1 mA, the current intensity increased by 2 mA until the current reached 8 mA or after discharges were recorded (whichever lower). Each session consisted of sixty stimuli with maximum intensity stimuli being delivered twice to confirm reproducibility. High and low filters for CCEPs of stimulus onset were 300 Hz and 1 Hz, respectively. Matlab (The Mathworks Inc., Natick, MA, USA), with the addition of a custom script, was used to display averaged CCEP waveforms by root mean square (RMS) of CCEP response between 10 and 500 ms post-stimulation to avoid weighing in stimulus-artifact.

fMRI data acquisition

Every subject had 6 runs of resting-state fMRI using a 3T MR scanner (Siemens Trio, Siemens Medical, Erlangen, Germany) with short breaks between runs (less than a minute) to provide high-quality data free of motion artifacts. Images were acquired with an echoplanar imaging sequence with a 2-second repetition time (TR), 30-millisecond echo time (TE), 256-mm field of view (FoV), 90° flip angle, 64 X 64 matrix size, 4 X 4 mm in-plane resolution, and 4-mm slice thickness without gap, generating 220 image volumes. Anatomical images were acquired with 2-dimensional (300-millisecond TR, 2.43-millisecond TE, 60° flip angle) and 3-dimensional T1-weighted sagittal magnetization-prepared rapid acquisition with a gradient echo (MPRAGE) sequence (2,530-millisecond TR, 3.34-millisecond TE, 7° flip angle) with 256-mm FoV, 256 X 256 matrix size, 1-mm thickness, and 176 contiguous slices.

fMRI network analysis

To calculate the intrinsic connectivity contrast–degree (ICC-d_th_) measure, the correlation between the rs-fMRI BOLD time course of a reference voxel centered around the mid-distance point between the two contacts evoking time dilation was, and all other gray matter voxels were determined.

1. Alkawadri R, Gaspard N, Goncharova, II, et al. The spatial and signal characteristics of physiologic high frequency oscillations. *Epilepsia* 2014;55(12):1986-95. doi: 10.1111/epi.12851 [published Online First: 2014/12/04]

2. Kucewicz MT, Cimbalnik J, Matsumoto JY, et al. High frequency oscillations are associated with cognitive processing in human recognition memory. *Brain* 2014;137(Pt 8):2231-44. doi: 10.1093/brain/awu149 [published Online First: 2014/06/13]

3. Cohen MX. Analyzing neural time series data: theory and practice: MIT press 2014.

4. Seth AK, Barrett AB, Barnett L. Granger causality analysis in neuroscience and neuroimaging. *Journal of Neuroscience* 2015;35(8):3293-97.
